# Betaine: a promising novel anti-aging substance as an exercise mimetic

**DOI:** 10.3389/fphar.2025.1672934

**Published:** 2025-08-28

**Authors:** Yonghui Liu, Jiang Lin

**Affiliations:** College of Basic Medicine, Guangxi University of Chinese Medicine, Nanning, China

**Keywords:** exercise, betaine, TBK1, inflammation, aging

## Introduction

Exercise refers to planned, structured, and repetitive physical activities aimed at improving or maintaining health and physical fitness. Beyond enhancing physical fitness, exercise is widely recognized as a highly effective non-pharmacological intervention for preventing chronic diseases and mitigating age-related functional decline (van der Ploeg and Bull, 2020). In metabolic disorders such as type 2 diabetes, exercise enhances insulin sensitivity through GLUT4-mediated glucose uptake, thereby improving blood sugar regulation and reducing complications (Magkos et al., 2020). Similarly, for cardiovascular diseases, it strengthens cardiac contractility, improves vascular endothelial function, and optimizes lipid profiles, which in turn lowers the incidence of atherosclerosis and myocardial infarction (Tucker et al., 2022). Moreover, in neurodegenerative conditions like Alzheimer’s disease, exercise upregulates neurotrophic factors and accelerates β-amyloid clearance, thus safeguarding cognitive function (De la Rosa et al., 2020). Additionally, in chronic inflammatory diseases such as rheumatoid arthritis, it modulates immune responses by reducing pro-inflammatory cytokines and promoting anti-inflammatory macrophage polarization, thereby alleviating symptom severity (Li and Wang, 2022). Critically, the development and progression of these diseases are closely intertwined with the aging process: aging elevates the risk of their onset, while disease progression in turn exacerbates age-related functional decline. Thus, exercise holds significant importance in anti-aging.

**FIGURE 1:**
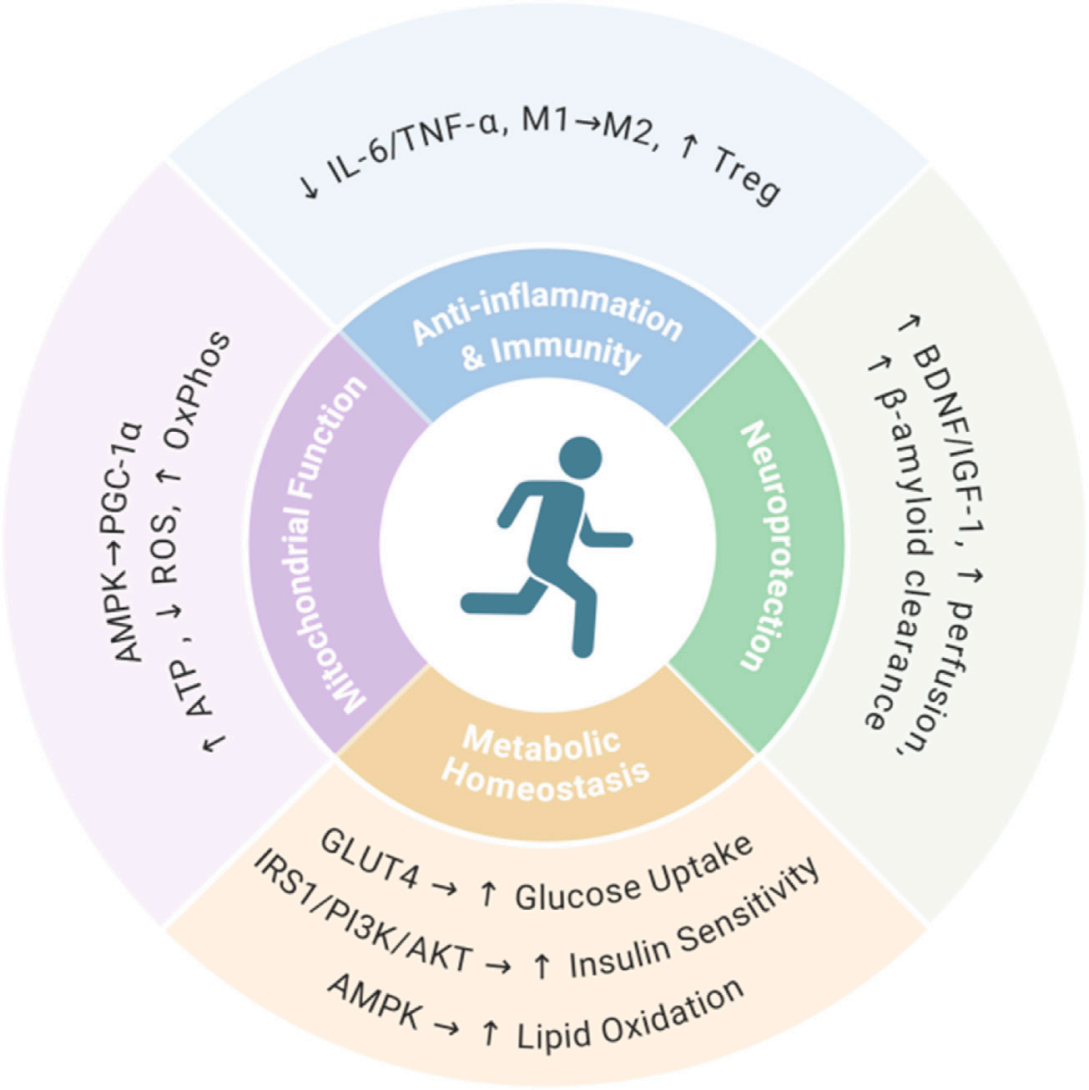
Core exercise-induced anti-aging mechanisms.

The anti-aging effects of exercise are mediated through multiple interconnected biological mechanisms (Lu et al., 2025; Lopez-Otin et al., 2023). Firstly, exercise enhances mitochondrial function via AMPK/PGC-1α activation, boosting biosynthesis efficiency and oxidative phosphorylation capacity while increasing ATP production and suppressing reactive oxygen species accumulation (Cartee et al., 2016; Hood et al., 2011). Secondly, exercise exerts anti-inflammatory and immunomodulatory effects by reducing proinflammatory cytokines (IL-6, TNF-α), promoting M2 macrophage polarization, and enhancing regulatory T cell function (Franceschi et al., 2018; Pangrazzi and Meryk, 2024). Thirdly, exercise provides neuroprotection through upregulated neurotrophic factors (BDNF, IGF-1), improved cerebral perfusion, and accelerated β-amyloid clearance (Cotman et al., 2007; Voss et al., 1985). Finally, exercise maintains metabolic homeostasis via GLUT4-mediated glucose uptake, IRS1/PI3K/AKT-dependent insulin sensitivity enhancement, and AMPK-coordinated lipid metabolism (Richter and Hargreaves, 2013; Stanford and Goodyear, 2016; Lehnig and Stanford, 2018) (Figure 1). Despite these multifaceted benefits, elderly individuals often struggle to maintain exercise due to physical decline, comorbidities, or environmental constraints. This limitation underscores the need for exercise-mimetic interventions to harness the anti-aging benefits of physical activity.

Recently, Geng et al. identified the renal metabolite betaine as a potent exercise mimetic through comprehensive multi-omics analysis of exercise responses, offering a promising solution for individuals unable to sustain long-term exercise (Geng et al., 2025). Specifically, through multi-omics analysis of 13 healthy males, Geng et al. systematically characterized the differential responses to Acute exercise (AE) and Long-term exercise (LE). AE primarily induced acute metabolic and immune stress, marked by significant increases in non-esterified fatty acids, decreased total bile acids, and upregulation of inflammatory factors including IL-6 and EN-RAGE, alongside activation of the glucocorticoid receptor pathway and enhanced anaerobic glycolysis. In contrast, LE triggered sustained adaptations involving metabolic reorganization, immune remodeling, and gut microbiota restructuring. Metabolic reorganization is achieved through the coupling of fatty acid oxidation with tricarboxylic acid cycle activity, accompanied by optimized amino acid metabolism and activated antioxidant defenses. Immune remodeling is reflected by increased naive lymphocytes, reduced neutrophils, and attenuated lymphocyte aging via ETS1 downregulation. Gut microbiota restructuring is characterized by a decrease in opportunistic pathogens and suppressed lipopolysaccharide biosynthesis. Critically, LE also specifically activated methionine metabolism pathways, inducing significant enrichment of the renal metabolite betaine.

**FIGURE 2:**
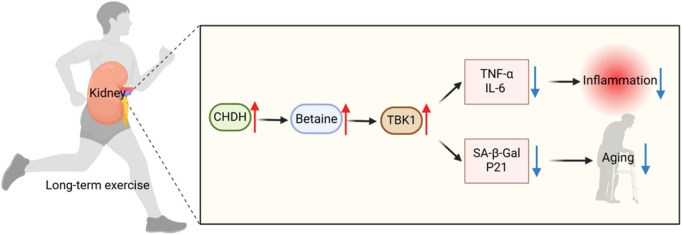
Mechanisms of long-term exercise (LE) against inflammation and aging. LE upregulates renal choline dehydrogenase (CHDH) expression to promote betaine biosynthesis. Betaine further inhibited the innate immunity key kinase TBK1, significantly reduced the release of pro-inflammatory factors TNF-α and IL-6, and reduced the levels of senescence markers SA-β-Gal and p21, thereby inhibiting inflammation and delaying aging.

Integrated multi-omics analysis confirmed the kidney as the central organ for exercise-induced betaine metabolism, mediated by upregulation of renal choline dehydrogenase (CHDH). Mechanistic studies demonstrated betaine directly binds and inhibits the innate immune kinase TBK1, reducing lipopolysaccharide-induced release of pro-inflammatory cytokines TNF-α and IL-6 while inhibiting immune cell adhesion. Murine models further established betaine’s capacity to alleviate cellular senescence, consistently reducing established aging markers including SA-β-Gal and p21. These data reveal the kidney-betaine-TBK1 axis as the core pathway coordinating exercise-mediated anti-inflammatory and anti-senescence effects (Figure 2).

To validate the therapeutic efficacy of betaine, Geng et al. conducted comprehensive supplementation studies in aged murine models. They supplemented aged mice with 1% betaine daily for 8 weeks and found that betaine concentrations in the kidneys of aged mice increased to levels comparable to those induced by LE. Functional evaluation showed that aged mice had significantly improved motor coordination, muscle strength and spatial memory ability, and significantly reduced depression-like behaviors. Histopathological analysis revealed attenuated markers of aging, reduced lipid deposition, and reduced fibrosis in the kidney, liver, lung, and skin, along with restoration of skeletal muscle morphology and epidermal architecture. Molecular analysis confirmed that betaine could inhibit the phosphorylation of TBK1/IRF3/p65 signaling pathway, down-regulate the proinflammatory cytokines TNF-α and IL-1β, and activate AMPK/SIRT1/PGC-1α signaling pathway. These collective findings support betaine as a viable exercise-mimetic intervention for counteracting age-related physiological and functional decline.

However, we recognize that this study has certain limitations. The human cohort comprised only 13 healthy young males, representing both a small sample size and the exclusion of female participants. Preclinical validation was similarly restricted to aged male murine models. This male-exclusive design restricts generalizability to females, particularly given documented sex differences in hormonal regulation, betaine metabolic kinetics, and female-specific aging processes. Crucially, betaine’s metabolism in female physiology and its impact on reproductive systems remain uncharacterized. We therefore emphasize the necessity for expanded investigations with balanced gender representation and larger cohorts to comprehensively evaluate betaine’s effects across diverse populations.

We propose that betaine’s most immediate translational value lies in overcoming exercise adherence barriers in elderly populations. Although regular exercise significantly delays aging, elderly individuals frequently struggle with sustained physical activity due to age-related physical decline, comorbidities, or environmental constraints (Mora and Valencia, 2018). Betaine recapitulates exercise-mediated protection against multisystem aging: preserving neurocognitive function by suppressing microglial overactivation, combating sarcopenia through increased muscle fiber cross-sectional area, and reducing metabolic disease risk via improved glucose-lipid homeostasis (Geng et al., 2025). To advance clinical implementation, we recommend prioritizing long-term safety assessments and dose-response validation in elderly cohorts, coupled with exploration of synergistic formulations combining betaine with senolytic agents. These initiatives constitute our strategy to establish betaine as a viable non-pharmacological alternative for healthy aging promotion.

At the same time, we believe that betaine can be used as a fundamental candidate for ovarian aging intervention research. Ovarian aging constitutes a core manifestation of declining female reproductive and endocrine function, characterized by diminished follicular reserve, granulosa cell senescence, and chronic inflammatory microenvironments (Cavalcante et al., 2023). Aberrant TBK1/NF-κB signaling accelerates ovarian aging by promoting follicular atresia and granulosa cell apoptosis (Geng et al., 2025; Zhang et al., 2021). Given betaine’s specific inhibition of TBK1-mediated pro-inflammatory cascades, we propose investigating its capacity to mitigate ovarian inflammation and reduce senescence markers in granulosa cells. Subsequent development of betaine-based therapeutics could provide targeted alternatives to traditional hormone replacement for premature ovarian insufficiency and menopausal syndrome, potentially circumventing estrogen-associated comorbidities while restoring endocrine homeostasis.
